# Development and validation of a novel nomogram for recurrent hemoptysis after bronchial artery embolization: a population-based cohort study

**DOI:** 10.3389/fmed.2025.1705253

**Published:** 2025-12-19

**Authors:** Jing Yu, Lei Qin, Wei Li, Wen-Ze Wu, Ji-Dong Yang, Mao-Lin Wan, Xiao-Long Li, Wan-Yao Zhang, Jin-Ke Huang, Qing-Ao Xiao, Xiao-Lin Zhang

**Affiliations:** 1 Department of Interventional Radiology, The First College of Clinical Medical Science, China Three Gorges University, Yichang, Hubei, China; 2 Department of Interventional Radiology, Jingmen People's Hospital, JingChu University of Technology Affiliated Central Hospital, Jingmen, Hubei, China; 3 Department of Interventional Radiology, Jingzhou Central Hospital, Jingzhou Hospital Affiliated to Yangtze University, Jingzhou, Hubei, China; 4 Department of Radiology, Xiangya Hospital, Central South University, Changsha, Hunan, China

**Keywords:** hemoptysis, bronchial artery embolization, hemoptysis recurrence, clinical predictive model, nomogram

## Abstract

**Background and objective:**

Recurrent hemoptysis after bronchial artery embolization (BAE) remains a significant clinical challenge. This study aims to develop and validate a predictive model to forecast hemoptysis recurrence post-BAE, enhancing clinical decision-making.

**Methods:**

A retrospective analysis was conducted on 170 patients with hemoptysis from various causes who underwent their first BAE at three Chinese medical centers between January 2019 and December 2022. Data were split into training and validation groups (7:3 ratio). Independent predictors for recurrence were identified using the least absolute shrinkage and selection operator and multivariable logistic regression. Three models were developed: clinical (Model C), radiological (Model R), and combined (Model CR). The models’ performance was evaluated via receiver operating characteristic (ROC) curves, net reclassification improvement (NRI), integrated discrimination improvement (IDI), and calibration curves, to determine the best model. Decision curve analysis (DCA) was used to assess clinical benefits. A nomogram was then created using the optimal model.

**Results:**

Independent predictors for recurrence included clinical factors (hemoptysis volume, platelets, C-reactive protein) and radiological factors (fibrotic scarring, pleural thickening, bronchial artery diameter, number of arteries). Based on these, Model C and Model R were created, and Model CR incorporated all seven factors. Model CR outperformed the other models, with superior accuracy in both the training cohort (AUC: 0.931, 95% CI: 0.864–0.998) and validation cohort (AUC: 0.883, 95% CI: 0.792–0.974). Comparative tests (DeLong test, NRI, and IDI) showed Model CR had better predictive power. Calibration curves and the Hosmer-Lemeshow test confirmed good model fit (*P*_H-L_ > 0.05). DCA revealed Model CR provided the most clinical benefit. A nomogram was developed from Model CR.

**Conclusion:**

The nomogram based on clinical and radiological data shows strong predictive accuracy for hemoptysis recurrence after BAE, offering significant potential for clinical integration.

## Introduction

Hemoptysis is one of the most common clinical symptoms of the respiratory system, typically occurring in diseases such as tuberculosis (TB), bronchiectasis, and pulmonary tumors (1). Mild to moderate hemoptysis generally has a good prognosis; however, massive hemoptysis can lead to asphyxia or severe blood loss, posing a life-threatening risk to patients (2). Bronchial artery embolization (BAE) is a safe and effective method for managing hemoptysis (3, 4). In a systematic review and meta-analysis, Fan et al. found that the clinical success rate of BAE in treating hemoptysis was 92.2%, while the hemoptysis-related mortality rate was only 0.8% (5). However, BAE is not a curative procedure and cannot directly address the underlying cause of hemoptysis. After undergoing BAE, the recurrence rate of hemoptysis remains relatively high, ranging from 9.8 to 57.5%, and the median interval until recurrence is approximately 6 months to 1 year (6).

The recurrence of hemoptysis following BAE has emerged as a critical concern that must be addressed in the management of this condition. It is generally believed that early recurrence of hemoptysis within 1 month after BAE is primarily due to incomplete embolization of the responsible vessel (7, 8). As for the long-term recurrence beyond 1 month, current studies have not reached a consensus on the exact causes, though it is thought to be related to recanalization of the embolized vessels and the formation of new bleeding vessels (9). Several recent studies have investigated the causes of long-term hemoptysis recurrence after BAE, identifying related risk factors from the perspectives of underlying conditions, the volume of hemoptysis, and other contributing factors (3, 10–12). However, these studies have only reported risk factors for hemoptysis recurrence without addressing the weight or relative importance of different risk factors in the recurrence process, which limits the clinical applicability for patients with hemoptysis.

Earlier, Yan et al. predicted recurrence of hemoptysis after BAE in patients with bronchiectasis and non-tumor-related hemoptysis, but did not include tumor-related hemoptysis in their study, which limited the generalizability of their findings (13, 14). Similarly, Xu et al. developed a novel artificial neural network model to predict hemoptysis recurrence after BAE, but like the study by Yan et al., this research did not differentiate between short-term recurrence within 1 month and long-term recurrence (15). Furthermore, the extended follow-up periods in both studies may have been problematic, as the underlying causes of hemoptysis could change over time during follow-up (16).

To better predict long-term hemoptysis recurrence after BAE and provide more precise guidance for the clinical management of relevant cases, this study conducted a large-scale, retrospective, multi-center cohort analysis. This study established and validated multiple clinical predictive models, ultimately selecting the optimal model to construct a nomogram. This model is capable of accurately predicting hemoptysis recurrence within 1 month to 2 years after BAE, thereby assisting clinicians in providing precise preventive and therapeutic strategies for patients post-BAE.

## Materials and methods

### Study design

**Figure 1:**
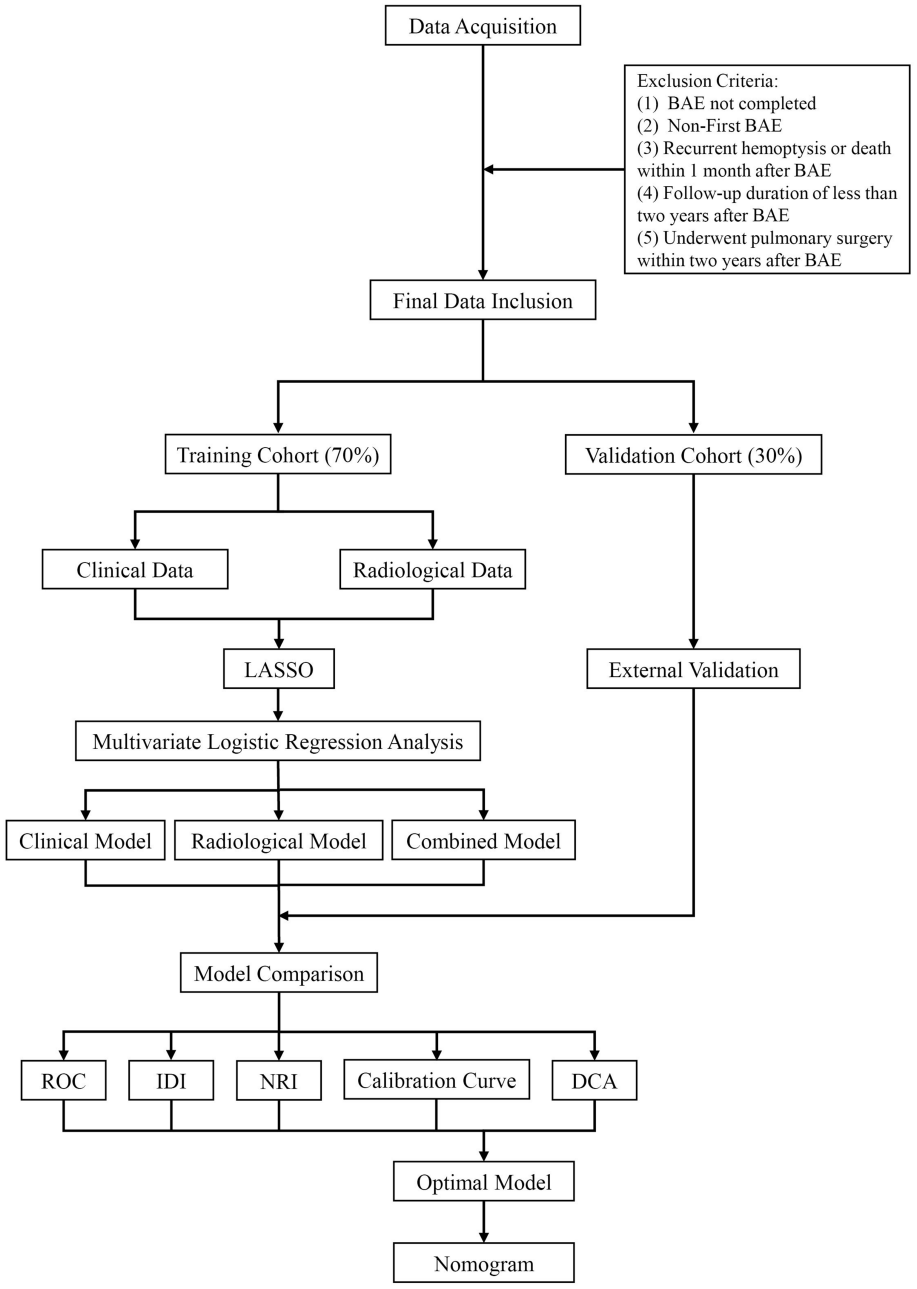
Flowchart of the research process. BAE, bronchial artery embolization; LASSO, least absolute shrinkage and selection operator; ROC, receiver operating characteristic; IDI, integrated discrimination improvement; NRI, net reclassification index; clinical decision curves.

This study retrospectively collected patient data from three medical centers in China between January 2019 and December 2022. Patients who were first treated with BAE for hemoptysis of various etiologies were identified using the hospitals’ electronic medical record systems. After identifying patients with hemoptysis who met the inclusion criteria, individuals who met the exclusion criteria were excluded. The remaining eligible patients were randomly allocated into two groups: a training cohort comprising 70% of the sample and an internal validation cohort consisting of 30%. Data from the training cohort were used to construct models for hemoptysis recurrence after BAE, while the internal validation cohort was used to assess whether the model’s predictive performance in the internal dataset was consistent with its performance in the training cohort. In this study, we developed three predictive models: a clinical model (Model C), a radiological model (Model R), and a combined model (Model CR). These models were constructed through the following steps: (1) clinical and radiological data were separately subjected to least absolute shrinkage and selection operator (LASSO) regression and multivariable logistic regression analyses to identify independent predictors specific to each data source; (2) based on the identified independent predictors, the clinical model, radiological model, and combined model were subsequently established. The models were evaluated through various metrics, including receiver operating characteristic (ROC) curves, net reclassification index (NRI), integrated discrimination improvement (IDI), and calibration curves. In addition, the models’ predictive accuracy was confirmed through internal validation using the data from the internal validation cohort. After identifying the optimal model among the three BAE-related hemoptysis recurrence prediction models, clinical decision curves (DCA) for all models were constructed to assess the additional benefits they could bring to patients. Finally, a nomogram for the best model was developed. Figure 1 illustrates a flowchart that details the process of the study. Subgroup analysis was performed to explore the distribution of independent predictors across different etiological subgroups. Additionally, interaction analysis was conducted using multivariable logistic regression to evaluate whether the association between platelets (PLT, a key independent predictor) and hemoptysis recurrence varied by etiology. This study adhered to the guidelines outlined in the Transparent Reporting of a Multivariable Prediction Model for Individual Prognosis or Diagnosis (TRIPOD) statement, with compliance verified using the TRIPOD checklist (Supplementary Table S1).

### Data acquisition

This retrospective multicenter study collected data from 285 patients who underwent BAE for hemoptysis due to various etiologies at three medical centers in China between January 2019 and December 2022. Patient information that met any of the following criteria was excluded from this study: (1) BAE not completed, (2) Non-first BAE, (3) Recurrence or death within 1 month after BAE, (4) Follow-up time after BAE was less than 2 years, (5) Received pulmonary surgery within 2 years after BAE. The data were primarily categorized into two types: clinical data and radiological data. To minimize human bias, clinical information and laboratory test data were collected independently by two individuals. If any discrepancies arose, a third person was brought in to verify and resolve the discrepancy. For the radiological data, the average measurements from two individuals were selected. To ensure the reliability of radiological measurements, inter-observer consistency was assessed for all radiological indicators. For categorical variables, including fibrotic scar, cavity, pleural thickening, and systemic artery-pulmonary artery fistula, Cohen’s Kappa coefficient was used, with a Kappa value > 0.75 defined as good consistency. For continuous variables, namely maximal bronchial artery diameter (MBAD) and number of bronchial arteries (NBA), the intraclass correlation coefficient (ICC) was calculated, with an ICC > 0.70 considered acceptable consistency.

The clinical data were primarily derived from the admission records and blood test results during hospitalization. These data include the following information: (1) General patient information: gender, age, etiology of hemoptysis, history of diabetes, history of hypertension, history of pulmonary surgery before BAE, smoking history, history of hemoptysis, amount of hemoptysis, duration of hemoptysis, and recurrence of hemoptysis after BAE. (2) Laboratory blood test data: hemoglobin (Hb), white blood cells (WBC), neutrophils, platelets (PLT), C-reactive protein (CRP), prothrombin time (PT), and international normalized ratio (INR). Laboratory test results were required to be obtained within 7 days before BAE. In cases where multiple tests for the same parameter were performed, the latest result obtained before the procedure was utilized. Additionally, the history of pulmonary surgery was defined as any non-hemoptysis-related lung surgery performed before BAE. Hemoptysis volume was classified into three categories: massive hemoptysis was characterized by a volume of blood expectorated exceeding 300 mL within 24 h or more than 100 mL within 1 h; moderate hemoptysis was classified as expectoration of blood ranging from 100 to 300 mL within 24 h; minor hemoptysis was characterized by the expectoration of blood amounting to less than 100 mL (7, 17). The duration of hemoptysis was measured from the initial episode of hemoptysis until the patient underwent BAE treatment. Long-term recurrence of hemoptysis was defined as a single-day hemoptysis volume of 10 mL or more 1 month after BAE treatment (10).

The radiological data were primarily obtained from high-resolution computed tomography (HRCT), bronchial artery computed tomography angiography (CTA) performed within 7 days before BAE, and intraoperative surgical information during BAE. The data included the following information: (1) General pulmonary condition: pulmonary fibrosis, cavity, and pleural thickening. (2) Pulmonary vascular status: MBAD, NBA, and systemic arterial-pulmonary artery fistula. (3) Intraoperative surgical information during BAE: number of embolized bronchial arteries, number of embolized nonbronchial systemic arteries (NBSA), and embolic materials. HRCT imaging was used to assess the general pulmonary condition of the patient, including the presence of pleural thickening, cavity, and fibrotic scar. Pleural thickening was identified when the pleural thickness on the affected side of the lung exceeded 3 mm as observed on preoperative HRCT scans (18). A cavity is defined as a round or ovoid hypodense area identified in the lesional region of the affected lung on HRCT, characterized by well-defined cavity walls and may be accompanied by fluid levels and mural nodules (19). Fibrotic scar is defined as follows: on preoperative HRCT, they present as well-defined linear or patchy high-density opacities in the affected side of the lungs, may be accompanied by lung structure distortion, with the exclusion of acute inflammatory exudation (20). Bronchial artery CTA prior to BAE is used to evaluate the type of bleeding vessels, the number, and maximal diameter of bronchial arteries, as well as systemic arterial-pulmonary artery fistulas. The diameter measurement of MBAD is performed by selecting, on bronchial artery CTA images with a slice thickness = 1 mm, either the main trunk of the bronchial artery within 1 cm of its origin from the descending aorta or unbranched first-order branches in the perihilar region (21). Vessels leading to the hemorrhagic lesion were considered the target bleeding vessels. When it was difficult to identify the target bleeding vessel or systemic arterial-pulmonary artery fistula on the CTA, additional angiography of the bronchial arteries, subclavian arteries, intercostal arteries, and diaphragmatic arteries was performed to fully identify the target bleeding vessels and systemic arterial-pulmonary artery fistula. After the bleeding source and systemic arterial-pulmonary artery fistula were located in all patients included in the study, the responsible bleeding vessels were completely embolized, and the fistula orifice of the systemic arterial-pulmonary artery fistula was fully occluded. The embolic agents used comprised polyvinyl alcohol (PVA) particles and PVA embolization microspheres. The size of the embolic material was chosen according to the diameter of the target vessel as observed in the angiographic images. Furthermore, all embolic materials were delivered into the target vessels via microcatheter super-selection. The criteria for confirming successful embolization were that post-procedural angiography confirmed the occlusion of the arteries and the absence of residual blood flow at the lesion site.

### Statistical analysis

Data processing and analysis were carried out using R software, version 4.2.3. Based on the data distribution, continuous variables were reported as either the mean ± standard deviation (SD) or the median with interquartile range (IQR). Categorical variables were expressed as numbers and percentages. Continuous variables were assessed using either the independent two-sample *t*-test or the Wilcoxon rank-sum test, whereas categorical variables were evaluated with the Pearson chi-square test or Fisher’s exact test. In the analysis, a *p*-value below 0.05 was regarded as indicative of a statistically significant difference between the groups.

### Predictor screening

The study employed the LASSO algorithm, in conjunction with multivariable logistic regression, to identify the predictors for the recurrence of hemoptysis after BAE, using data from the training cohort. Given the presence of interrelated variables (such as coagulation-related indicators like PT and INR), and the unique advantage of LASSO regression in handling high-dimensional covariate structures, this approach was utilized to mitigate the risk of model overfitting (22). LASSO regression provides two variable selection cutoff points, λ_min_ and λ_1SE_. λ_min_ represents the value of λ at the best fit in the LASSO regression selection, while λ_1SE_ represents the value of λ at one standard error to the right of λ_min_. Drawing from existing literature, this study chose variables at λ_min_ for multivariable logistic regression analysis and identified those with a *p*-value below 0.05 as independent predictors for the recurrence of hemoptysis after BAE (23). In the process of predictor selection, clinical and radiological data were independently analyzed to identify respective predictors. Subsequently, separate models were constructed based on these predictors, and a combined model was built using all the predictors derived from both sets of data.

### Model development and comparison

Three models (Model C, Model R, and Model CR) for post-BAE hemoptysis recurrence were constructed according to the data sources. After constructing the models, the ROC curve and the area under the curve (AUC) were utilized to assess and contrast the discriminatory ability of the three models. The DeLong test was then applied to assess if there were significant differences in the AUC values between the three models (24). Subsequently, the NRI and IDI were used to compare the relative strengths and weaknesses of the three models. The NRI specifically measures the change in predictive accuracy between the new and old models, distinguishing between patients with recurrence and those without. An NRI greater than 0 indicates that the new model outperforms the old one, while an NRI less than 0 suggests inferior performance (25). An NRI of 0 means there is no significant difference in prediction accuracy between the two models (25). IDI evaluates the disparity in predicted probabilities between the new and old models. Like the NRI, a positive IDI suggests superior performance of the new model, while a negative value favors the old model, and an IDI of zero signifies that there is no significant distinction in the performance of the two models (26). Next, the calibration curve was employed to evaluate how well the model’s predictions align with the actual outcomes. Additionally, the Hosmer-Lemeshow (H-L) test was used to evaluate if a statistically significant difference existed between the two (27). A *P_H-L_* value less than 0.05 suggests a statistically significant difference between the model’s predicted outcomes and the actual results, indicating a poor prediction performance. Conversely, a *P_H-L_* value greater than 0.05 implies no significant difference, suggesting strong prediction accuracy. Finally, DCA was employed to assess the clinical value of applying the models to patients (28). After completing the model comparison, the one that outperformed the others in all evaluation metrics was chosen as the optimal model.

### Model visualization

Following the selection of the optimal model, a nomogram was developed to support clinicians in determining the scores associated with each predictor for the patient. Once the scores for all predictors are obtained and summed, the total score can be used to calculate the probability of hemoptysis recurrence in patients after BAE.

## Results

### Baseline characteristics

Data were gathered from 285 patients who received BAE as a treatment for hemoptysis in this study. After screening, 115 patients who did not meet the inclusion criteria were excluded, leaving a total of 170 patients with hemoptysis of various etiologies who received their first BAE treatment. Among the 115 excluded patients, 2 had incomplete BAE procedures, 9 had non-first BAE procedures, 39 experienced recurrence or death within 1 month after BAE, 56 had insufficient follow-up (less than 2 years) after BAE, and 9 underwent pulmonary surgery within 2 years following BAE.

A random allocation method was used to assign all patients to either the training cohort (*n* = 118, 70%) or the internal validation cohort (*n* = 52, 30%). There were no notable differences in baseline characteristics between the two groups (all *p* > 0.05) as shown in Table 1. Of all the patients, 120 (71%) were male and 50 (29%) were female, with the median age being 63 years. The three leading potential causes of hemoptysis were isolated bronchiectasis (46%), mixed etiology (combinations of any two or more of bronchiectasis, TB, and pulmonary malignancies) (25%), and isolated pulmonary malignancy (15%).

**Table 1:** The baseline characteristics of hemoptysis patients undergoing bronchial artery embolization.

Variables	Total (*n* = 170)	Training cohort (*n* = 118)	Validation cohort (*n* = 52)	*p* value
Gender, *n* (%)				0.242
Male	120 (71)	87 (74)	33 (63)	
Female	50 (29)	31 (26)	19 (37)	
Age, Median (Q1, Q3)	63 (55, 70)	63 (55, 68.75)	62.5 (55, 71)	0.772
Etiology, *n* (%)				0.998
Bronchiectasis	79 (46)	54 (46)	25 (48)	
TB	6 (4)	4 (3)	2 (4)	
Malignancy	25 (15)	18 (15)	7 (13)	
Mixed etiology	43 (25)	30 (25)	13 (25)	
Others	17 (10)	12 (10)	5 (10)	
Diabetes, *n* (%)				0.471
No	153 (90)	108 (92)	45 (87)	
Yes	17 (10)	10 (8)	7 (13)	
Hypertension, *n* (%)				0.762
No	122 (72)	86 (73)	36 (69)	
Yes	48 (28)	32 (27)	16 (31)	
History of lung surgery, *n* (%)				0.134
No	161 (95)	114 (97)	47 (90)	
Yes	9 (5)	4 (3)	5 (10)	
Smoking history, *n* (%)				0.451
No	94 (55)	68 (58)	26 (50)	
Yes	76 (45)	50 (42)	26 (50)	
Hemoptysis volume, *n* (%)				0.860
Minor	85 (50)	59 (50)	26 (50)	
Moderate	23 (14)	17 (14)	6 (12)	
Massive	62 (36)	42 (36)	20 (38)	
Hemoptysis duration, Median (Q1, Q3)	3 (0.5, 195)	2.5 (0.5, 208)	4 (0.5, 117)	0.771
History of hemoptysis, *n* (%)				0.237
No	115 (68)	76 (64)	39 (75)	
Yes	55 (32)	42 (36)	13 (25)	
Hb, Mean ± SD	123.01 ± 19.67	124.03 ± 20.87	120.69 ± 16.59	0.267
WBC, Median (Q1, Q3)	6.66 (5.33, 8.54)	6.38 (5.12, 8.5)	7.12 (5.59, 8.72)	0.123
PLT, Median (Q1, Q3)	161.5 (125, 210.75)	160 (128, 214)	166.5 (124, 206)	0.791
Neutrophils, Median (Q1, Q3)	4.88 (3.53, 6.97)	4.63 (3.42, 6.65)	5.64 (4.25, 7.41)	0.073
CRP, Median (Q1, Q3)	3.32 (1.1, 15.84)	3.21 (1.1, 16.02)	4.97 (1.12, 15.84)	0.866
PT, Median (Q1, Q3)	13.1 (12.22, 14)	13.1 (12.3, 14)	13.2 (12.2, 13.83)	0.826
INR, Median (Q1, Q3)	1.1 (1.03, 1.21)	1.1 (1.04, 1.22)	1.11 (0.99, 1.2)	0.301
Fibrotic scar, *n* (%)				1.000
No	150 (88)	104 (88)	46 (88)	
Yes	20 (12)	14 (12)	6 (12)	
Cavity, *n* (%)				0.458
No	161 (95)	113 (96)	48 (92)	
Yes	9 (5)	5 (4)	4 (8)	
Pleural thickening, *n* (%)				0.043
No	77 (45)	60 (51)	17 (33)	
Yes	93 (55)	58 (49)	35 (67)	
MBAD, Median (Q1, Q3)	3.2 (2.4, 3.6)	3.15 (2.4, 3.58)	3.2 (2.55, 3.65)	0.381
NBA, *n* (%)				0.629
1	15 (9)	10 (8)	5 (10)	
2	94 (55)	66 (56)	28 (54)	
3	51 (30)	37 (31)	14 (27)	
4	8 (5)	4 (3)	4 (8)	
5	2 (1)	1 (1)	1 (2)	
Embolic materials, *n* (%)				0.842
PVA only	141 (83)	99 (84)	42 (81)	
Microsphere only	3 (2)	2 (2)	1 (2)	
PVA + microsphere	26 (15)	17 (14)	9 (17)	
Systemic artery-pulmonary artery fistula, *n* (%)				0.460
No	135 (79)	96 (81)	39 (75)	
Yes	35 (21)	22 (19)	13 (25)	
Number of embolized BA, *n* (%)				0.787
1	34 (20)	25 (21)	9 (17)	
2	91 (54)	64 (54)	27 (52)	
3	40 (24)	26 (22)	14 (27)	
4	5 (3)	3 (3)	2 (4)	
Number of embolized NBSA, *n* (%)				0.080
0	110 (65)	81 (69)	29 (56)	
1	29 (17)	15 (13)	14 (27)	
2	19 (11)	14 (12)	5 (10)	
3	7 (4)	6 (5)	1 (2)	
4	5 (3)	2 (2)	3 (6)	
Recurrence, *n* (%)				0.118
No	135 (79)	98 (83)	37 (71)	
Yes	35 (21)	20 (17)	15 (29)	

TB, tuberculosis; mixed etiology refers to the presence of at least two causes among bronchiectasis, TB, and malignant tumors. Others include 1 case of pulmonary abscess, 4 cases of pulmonary infection, 1 case of pneumoconiosis, 3 cases of chronic obstructive pulmonary disease, 4 cases of vascular malformations, and 4 cases with unknown causes. Hb, hemoglobin; WBC, white blood cell; PLT, platelets; CRP, C-reactive protein; PT, prothrombin time; INR, international normalized ratio; MBAD, maximum bronchial artery diameter; NBA, number of bronchial arteries; PVA, polyvinyl alcohol; BA, bronchial artery; NBSA, nonbronchial systemic artery.

According to angiographic and arterial embolization results, more than half of the patients (54%) underwent embolization of two bronchial arteries, 60 patients (35%) had NBSA bleeding, 35 patients (21%) had pulmonary arteriovenous fistulas, and most patients (83%) received treatment with PVA embolization exclusively. During follow-up, 135 patients (79%) did not experience a recurrence of hemoptysis, while 35 patients (21%) experienced hemoptysis recurrence between 1 month and 2 years after BAE treatment.

### Inter-observer consistency of radiological indicators

All radiological indicators demonstrated good inter-observer agreement (Supplementary Table S2). For categorical indicators, Cohen’s Kappa coefficients ranged from 0.86 (pleural thickening) to 0.89 (cavity), with all *p*-values < 0.05. For continuous indicators, the ICCs for MBAD and NBA were 0.79 and 0.83, respectively, with both *p*-values < 0.05. These findings validate the reliability of radiological measurements, supporting their application in subsequent risk factor analyses.

### Predictors selection

**Figure 2:**
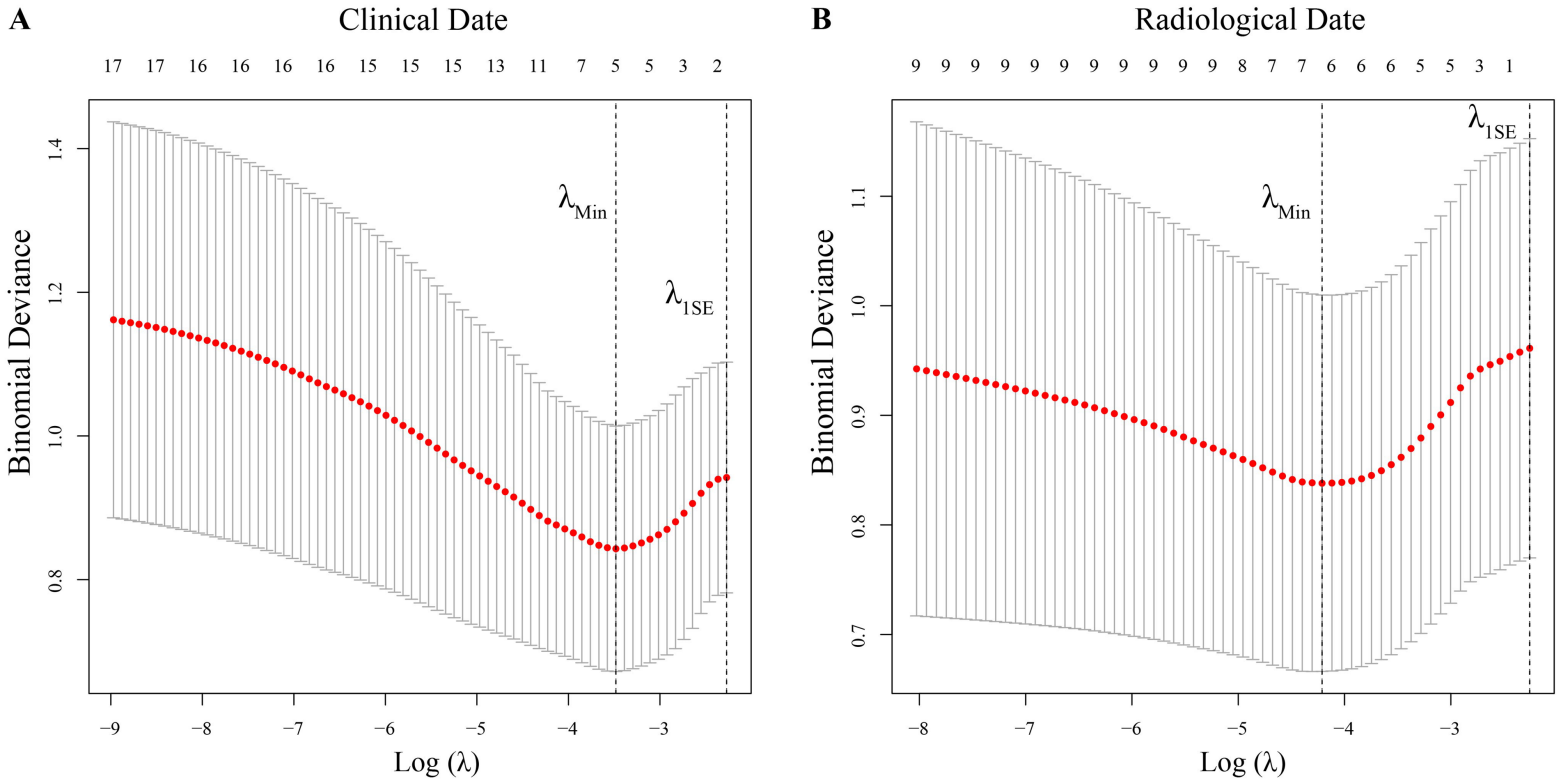
LASSO regression for variable selection. **(A)** LASSO regression analysis of clinical data; **(B)** LASSO regression analysis of radiological data. Use LASSO regression analysis to perform feature selection separately on clinical data and radiological data, and select the variables at the λ_min_ value as the selection result. Five variables were ultimately chosen from the clinical data, which are as follows: hypertension, hemoptysis volume, PLT, CRP, and PT. Six variables were eventually selected from the radiological data, which are as follows: fibrotic scar, cavity, pleural thickening, maximum bronchial artery diameter, number of bronchial arteries, and embolism of NBSA. λ_min_ represents the value of λ at the best fit in the LASSO regression selection, and λ_1SE_ represents the value of λ at one standard error to the right of λ_min._ The λ parameter in the LASSO regression analysis of clinical data is: λ_min_: 0.031, λ_1SE_ 0.103; The λ parameter in the LASSO regression analysis of radiological data is: λ_min_: 0.015, λ_1SE_ 0.105.

This study took into account a total of 26 feature variables. After grouping, the clinical group included 17 variables, and the radiological group included 9 variables. The training cohort data underwent LASSO analysis to determine the candidate predictors. The results of the LASSO analysis for both variable groups are displayed in Figure 2. Variables with non-zero coefficients at λ_min_ in the LASSO regression were chosen as potential associated factors. After performing LASSO regression analysis on clinical factors, five variables were selected (λ_min_: 0.031, λ_1SE_ 0.103), which were hypertension, hemoptysis volume, PLT, CRP, and PT (Figure 2A). Six variables were selected for radiological factors (λ_min_: 0.015, λ_1SE_ 0.105), which were fibrotic scar, cavity, pleural thickening, MBAD, NBA, and the number of embolized NBSA (Figure 2B).

Subsequently, multivariable logistic regression analysis was conducted on the selected candidate variables to identify the independent predictors for hemoptysis recurrence (Table 2). In this step, four variables, including hypertension, PT, cavity, and number of embolized NBSA, were excluded because the adjusted *p*-value was greater than 0.05. The study finally identified that hemoptysis volume (OR: 0.23, 95% CI 0.090–0.616), PLT (OR: 1.01, 95% CI 1.001–1.016), CRP (OR: 1.03, 95% CI 1.007–1.051), fibrotic scar (OR: 5.49, 95% CI 1.280–23.554), pleural thickening (OR: 5.99, 95% CI 1.649–21.750), MBAD (OR: 2.65, 95% CI 1.201–5.850), and NBA (OR: 0.30, 95% CI 0.119–0.767) are independent predictors for post-BAE hemoptysis recurrence.

**Table 2:** The multivariable logistic regression analysis of hemoptysis patients undergoing bronchial artery embolization.

Variables	OR	95%CI	*P* value
Clinical data			
Hemoptysis volume	0.23	0.090–0.616	0.003
PLT	1.01	1.001–1.016	0.018
CRP	1.03	1.007–1.051	0.010
Radiological data			
Fibrotic scar	5.49	1.280–23.554	0.022
Pleural thickening	5.99	1.649–21.750	0.006
MBAD	2.65	1.201–5.850	0.016
NBA	0.30	0.119–0.767	0.012

PLT, platelets; CRP, C-reactive protein; MBAD, maximum bronchial artery diameter; NBA, number of bronchial arteries.

### Model development and comparison

**Figure 3:**
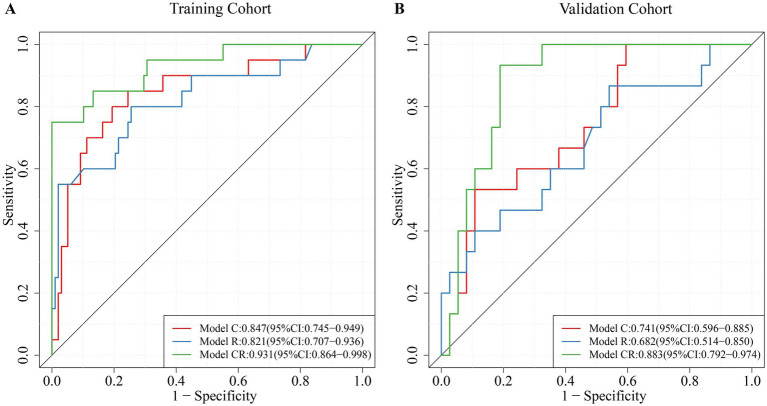
The ROC curves of the three models. **(A)** ROC curve of the training cohort; **(B)** ROC curve of the validation cohort.

Independent predictors were identified using multivariable logistic regression analysis, and these factors were then utilized to create two models: Model C and Model R. Additionally, Model CR was built using all seven independent predictors. ROC curves demonstrated that Model CR exhibited a higher AUC value in the training cohort (AUC: 0.931, 95% CI: 0.864–0.998) compared to the other two models (Model C: AUC: 0.847, 95% CI: 0.745–0.949; Model R: AUC: 0.821, 95% CI: 0.707–0.936, Figure 3). The conclusion was similarly applicable to the internal validation cohort (Model CR: AUC: 0.883, 95% CI: 0.792–0.974; Model C: AUC: 0.741, 95% CI: 0.596–0.885; Model R: AUC: 0.682, 95% CI: 0.514–0.850, Figure 3). The sensitivity, specificity, optimal cutoff point (Youden index), and AUC values for each model are presented in Table 3. Delong’s test was used to determine if the differences in AUCs were statistically significant, with results presented in Table 4. The analysis revealed significant differences between Model CR and Model C (*p* = 0.043), as well as between Model CR and Model R (*p* = 0.047), in the training cohort. These results were also consistent in the internal validation cohort. However, the comparison between Model C and Model R did not yield statistically significant differences in both the training and internal validation cohorts (*p* > 0.05).

**Table 3:** Comparison of sensitivity and specificity between different models.

Model	Sensitivity	Specificity	Youden index	AUC
Training cohort				
Model C	0.800	0.806	0.606	0.847
Model R	0.800	0.745	0.545	0.821
Model CR	0.750	1.000	0.750	0.931
Validation cohort				
Model C	0.533	0.892	0.425	0.741
Model R	0.867	0.459	0.326	0.682
Model CR	0.933	0.811	0.744	0.883

**Table 4:** Comparison of performance among different models.

Comparison	Delong test	NRI	*P _NRI_*	IDI	*P _IDI_*
Training cohort					
Model C vs. Model CR	0.043	1.110 (95%CI: 0.720–1.500)	<0.001	0.295 (95%CI: 0.164–0.427)	<0.001
Model C vs. Model R	0.768	−0.200 (95%CI: −0.673 to 0.273)	0.407	–	0.992
Model R vs. Model CR	0.047	1.231 (95%CI: 0.875–1.586)	<0.001	0.296 (95%CI: 0.190–0.402)	<0.001
Validation cohort					
Model C vs. Model CR	0.049	0.980 (95%CI: 0.454–1.506)	<0.010	0.189 (95%CI: 0.058–0.320)	0.005
Model C vs. Model R	0.679	−0.202 (95%CI: −0.799 to 0.396)	0.508	−0.060 (95%CI: −0.240 to 0.120)	0.516
Model R vs. Model CR	0.018	1.222 (95%CI: 0.745–1.699)	<0.001	0.248 (95%CI: 0.119–0.378)	<0.001

–: Unable to calculate; The NRI and IDI values obtained from the above model comparisons are derived by comparing the subsequent models with the previous ones. Therefore, if the NRI or IDI > 0, it indicates that the subsequent model performs better; conversely, if the values are less than 0, the opposite is true.

A comparison of the NRI and IDI across the models revealed that, in both the training and internal validation cohorts, the NRI and IDI values for Model CR versus Model C, and Model CR versus Model R, were positive, with *p*-values all under 0.05 (Table 4). These findings indicate that Model CR achieves superior performance compared with Model C and Model R in both prediction accuracy and probability estimation.

**Figure 4:**
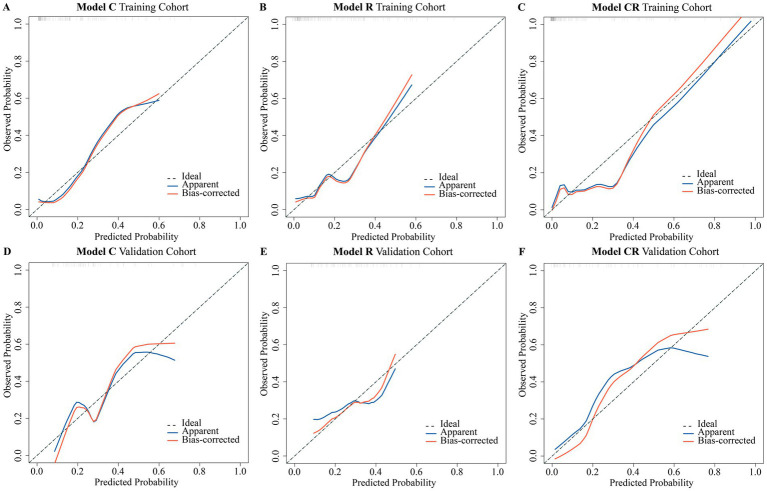
The Calibration curves of three models. **(A)** Calibration curve of Model C in the training cohort; **(B)** Calibration curve of Model R in the training cohort; **(C)** Calibration curve of Model CR in the training cohort; **(D)** Calibration curve of Model C in the validation cohort; **(E)** Calibration curve of Model R in the validation cohort; **(F)** Calibration curve of Model CR in the validation cohort. The dashed line represents the model’s predicted results that perfectly match the actual conditions, the blue line represents the model’s predicted results, and the red line represents the prediction results after 1,000 resamples.

To assess whether the predictive performance of the models aligns with actual outcomes, calibration curves were further constructed in this study (Figure 4). The H-L test results showed good fitting between the predicted and actual outcomes for all three models (*P_H-L_* > 0.05), with no statistically significant differences between them (Table 5).

**Table 5:** Hosmer-Lemeshow test for different models.

Model	Training cohort		Validation cohort	
	Statistic	*P* value	Statistic	*P* value
Model C	4.054	0.256	3.164	0.367
Model R	2.021	0.568	3.102	0.376
Model CR	5.030	0.170	3.931	0.269

Building on the above findings, this study observed that, across both the training and internal validation cohorts, Model CR significantly surpassed Model C and Model R in predicting post-BAE hemorrhage recurrence. As a result, Model CR was chosen as the optimal model for further research.

**Figure 5:**
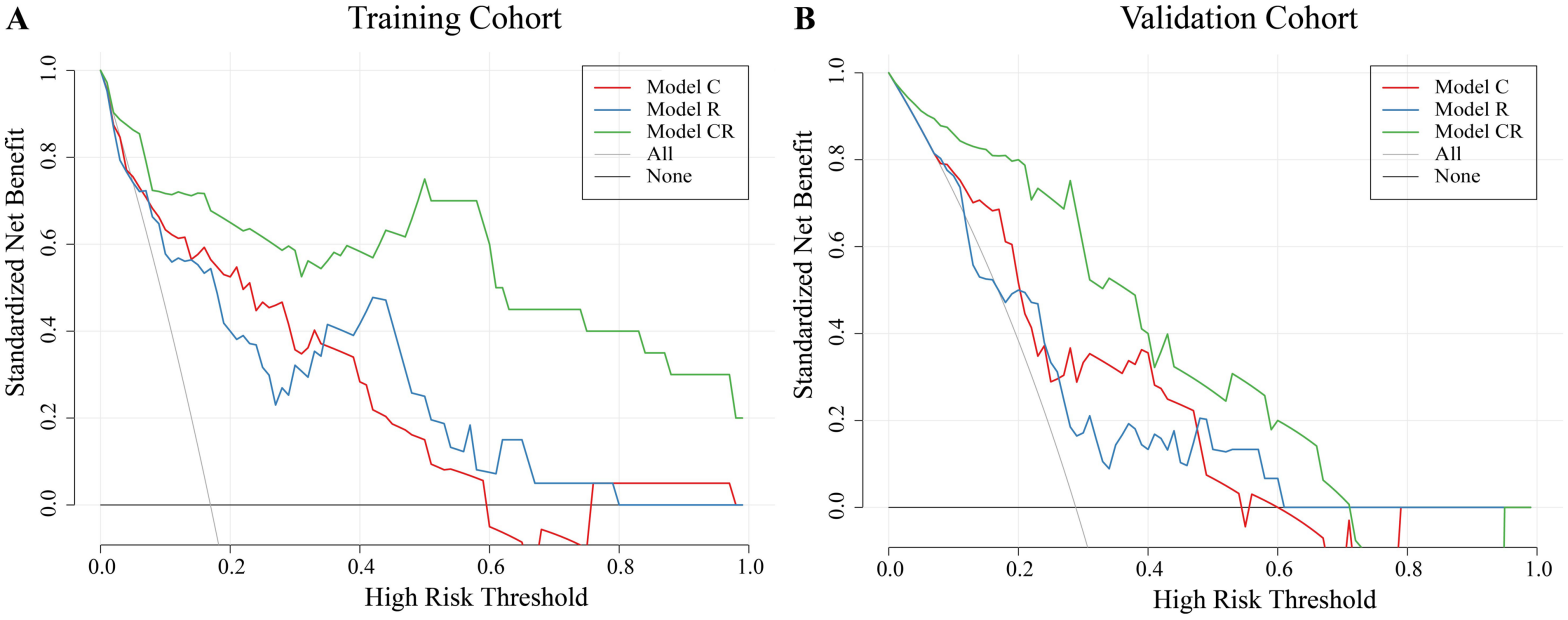
DCA curves of three models. **(A)** DCA curve of the training cohort; **(B)** DCA curve of the validation cohort.

The study then constructed DCA curves for all three models to assess the potential clinical benefits for patients. The DCA curves indicated that Model CR provided the greatest net benefit in both the training and internal validation cohorts, and the corresponding high-risk threshold range was the widest (Figure 5).

### Nomogram construction

**Figure 6:**
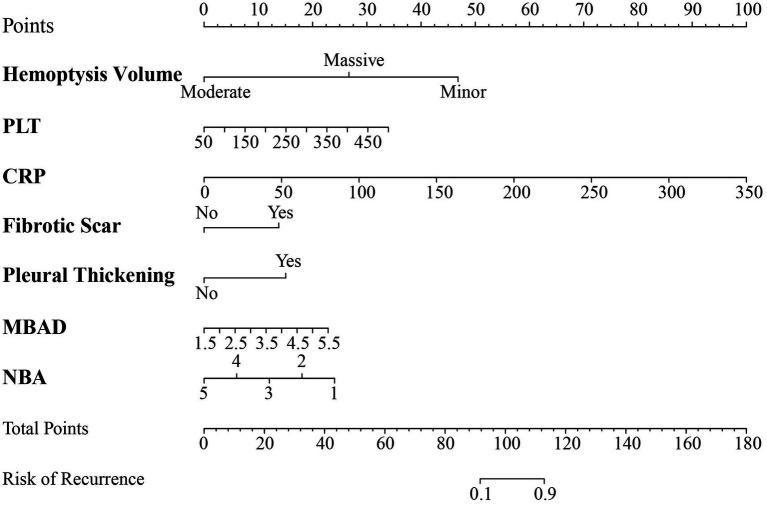
Nomogram development based on the optimal model. PLT, platelets; CRP, C-reactive protein; MBAD, maximum bronchial artery diameter; NBA, number of bronchial arteries.

Based on the aforementioned evaluations and comparisons, the optimal model, Model CR, was selected, and a nomogram was constructed (Figure 6). The nomogram effectively calculates the probability of rebleeding in a patient after undergoing BAE treatment within 1 month to 2 years, facilitating the development of personalized treatment plans for patients. Using the nomogram, scores for individual predictors such as hemoptysis volume, PLT, CRP, fibrotic scar, pleural thickening, MBAD, and NBA can be calculated. By summing these scores, the total score provides the risk of hemorrhage recurrence in patients within 1 month to 2 years after their first BAE treatment.

### Subgroup analysis of etiology and interaction analysis

Supplementary Table S3 shows the distribution of independent predictors and recurrence rates across etiological subgroups. There was no significant statistical difference in the six independent risk factors—CRP, hemoptysis volume, MBAD, NBA, fibrotic scar, and pleural thickening—as well as the hemoptysis recurrence rate among all etiological subgroups (bronchiectasis, TB, malignancy, mixed etiology, and others) (all *p* > 0.05). However, the PLT count was significantly higher in the malignancy subgroup (median 212 × 10^9^/L) than in the total cohort (median 161.5 × 10^9^/L; *p* = 0.014). To explore whether the predictive value of PLT varied by etiology, this study performed interaction analysis (Supplementary Table S4). The interaction term (PLT × etiology) was not significant (*p* = 0.834), indicating that the direction of PLT’s effect on recurrence was consistent across subgroups, and the overall model’s generalizability was not compromised.

## Discussion

With advancements in preoperative bronchial artery CTA, superselective coaxial microcatheter techniques, particulate embolization, and pre- and post-embolization angiography, the clinical and immediate success rates of BAE for hemoptysis have increased in recent years (29–31). As our study results show, the probability of hemoptysis recurrence within 2 years in 170 patients undergoing their first BAE treatment for hemoptysis was 21%, which aligns with the results reported by Sheehan et al. (30). However, it is important to note that the recurrence rate of hemoptysis after BAE has not significantly decreased over the past decade, remaining a key clinical challenge (32, 33). On one hand, BAE differs from surgical resection in that it can only control the symptoms of hemoptysis, rather than eliminate the underlying cause of the hemoptysis. On the other hand, although many studies have explored the causes of hemoptysis recurrence after BAE, no unified conclusion has been reached, adding complexity to the post-BAE follow-up care for patients. Previous studies have pointed out that short-term recurrence after BAE, especially recurrence within 1 month, is often due to incomplete embolization of the pathological vessels (8, 34). As for long-term recurrence after BAE, recent studies suggest that the peak of hemoptysis recurrence occurs within 2 years after the procedure (3, 35). However, there is still no consensus regarding the factors contributing to hemoptysis recurrence within 2 years after BAE, and no predictive models for this high recurrence period have been developed.

In this study, during the development of a model for hemoptysis recurrence from 1 month to 2 years after BAE, a total of 26 clinical and radiological characteristics were included to minimize the omission of potential risk factors. Seven independent predictors, including hemoptysis volume, PLT, CRP, fibrotic scar, pleural thickening, MBAD, and NBA, were identified and applied to build three models: Model C, Model R, and Model CR. Among these, Model CR was found to be the most effective after evaluation. Additionally, a nomogram was constructed based on all the independent predictors from Model CR.

To enhance the stability of the prediction model in this study, specifically to ensure the consistent predictive effect of the seven predictors on recurrence risk regardless of patients’ underlying etiologies, this study analyzed and tested the distribution differences of each predictor across different etiological subgroups. The results showed that among the seven predictors, only PLT count exhibited a statistically significant difference in distribution across etiological subgroups (*p* = 0.014 < 0.05). Subsequently, we analyzed the interaction effect between this predictor and different etiologies. The analysis results revealed a *p*-value for interaction = 0.834 (*p* > 0.05), indicating that the effect of PLT on recurrence risk did not differ significantly across etiological subgroups, which means there was no interaction effect between PLT and underlying etiologies. This result further confirms the generalizability of the prediction model in this study for application across different etiologies.

The seven independent predictors for hemoptysis recurrence after BAE identified in this study can be categorized into four main groups: pulmonary conditions (fibrotic scar and pleural thickening), laboratory blood indicators (PLT, CRP), vascular conditions (MBAD and NBA), and clinical manifestations (hemoptysis volume). Regarding the relationship between pulmonary conditions and hemoptysis recurrence, previous studies have indicated that pleural thickening is associated with the formation of non-bronchial system collateral vessels, which may contribute to hemoptysis recurrence after BAE (7). Building upon this, Yan H-T et al. provided additional evidence that pleural thickening serves as an independent risk factor for forecasting rebleeding (14). Although there has been no direct research showing that pulmonary fibrotic scar influences hemoptysis recurrence, lung fibrotic scarring may promote localized vascular abnormalities through inflammatory responses or fibrosis-related factors (36). Additionally, scarring leads to structural changes in lung tissue, which may affect the integrity of blood vessels and airways, increasing the risk of bleeding (37). A study by Sadidi S et al. further confirmed this conclusion, as their research demonstrated that destructive changes in lung tissue structure are independent predictors of hemoptysis recurrence after BAE (38).

In terms of the link between laboratory blood markers, like CRP and platelet count, and the recurrence of hemoptysis, previous studies have shown that CRP can inhibit the production of nitric oxide in vascular endothelial cells, weakening vascular repair capabilities and promoting the apoptosis of endothelial cells (39). These mechanisms may worsen the fragility of blood vessels, thereby heightening the likelihood of hemoptysis. In a study on hemoptysis recurrence after BAE in TB patients, survival curve analysis by Kim SW et al. indicated that individuals with higher preoperative CRP levels had a considerably shorter period without recurrence, whereas the recurrence risk was significantly lower in the group with normal CRP levels (40). Zhang et al. identified that high preoperative CRP is an independent risk factor for recurrent hemoptysis, suggesting its potential as a key marker for assessing rebleeding risk (41). CRP, a marker of acute inflammation, is commonly raised during infections or inflammatory conditions. In pulmonary diseases (such as TB or bronchiectasis), elevated CRP may reflect the activity of underlying conditions, with active lesions (such as persistent inflammation or uncontrolled infections) increasing the risk of rebleeding (41). Currently, no other studies have directly proven an association between platelet count and hemoptysis recurrence after BAE; however, platelet count directly affects coagulation function and may indirectly influence the recurrence of hemoptysis in post-BAE patients.

Regarding the relationship between the number and maximum diameter of bronchial arteries and hemoptysis recurrence, previous studies have suggested that a bronchial artery diameter of 3.5 mm or larger could be linked to a higher risk of severe hemoptysis (3). Furthermore, a larger bronchial artery diameter might result in a higher number of abnormal bronchial arteries. Hwang JH et al. demonstrated that bronchial artery abnormalities are significantly associated with recurrent hemoptysis (42). A study by Sadidi et al. on the predictive factors for hemoptysis recurrence after BAE explicitly stated that a bronchial artery diameter > 2 mm is an independent risk factor for hemoptysis recurrence after BAE (38). Consistent with the results of this study, all the aforementioned studies demonstrate that MBAD has an independent association with the recurrence of hemoptysis. Regarding the NBA, this study found that it is a protective factor against the recurrence of hemoptysis after BAE. This may be because a higher number of bronchial arteries allows for more comprehensive assessment of potential bleeding vessels during preoperative CTA and BAE, which is one of the potential factors for reducing the recurrence of hemoptysis. Another possible explanation is that the responsible blood vessels for hemoptysis are mostly bronchial arteries. When the number of bronchial arteries increases, the blood flow and intraluminal pressure of a single bronchial artery will decrease accordingly. This may reduce the probability of their re-rupture, thereby reducing the recurrence of hemoptysis.

This study also identified preoperative hemoptysis volume as an independent protective factor against hemoptysis recurrence. This finding differs from the conclusions of most post-BAE studies (30, 43). One potential explanation for this discrepancy is that massive hemoptysis, an acute condition, typically prompts more aggressive interventions, such as emergency BAE. Additionally, achieving complete control of massive hemoptysis requires a thorough vascular assessment and embolization of both bleeding vessels and potentially responsible ones. This may also contribute to the lower recurrence rate observed in patients with massive hemoptysis. However, it should be noted that this finding still needs further validation through studies with larger sample sizes.

There are some limitations in this study as well. Firstly, as a retrospective study, this study had limitations in patient data collection. For instance, this study only used permanent particulate embolic agents such as PVA and PVA microspheres, without including other materials. Previous studies have shown that using N-butyl cyanoacrylate (NBCA) for embolization resulted in one-year and three-year hemoptysis-free survival rates of 88 and 85%, respectively, with a recanalization rate of only 1.8% (44). Another retrospective study by Ishikawa H et al. demonstrated that embolization with metallic coils had a two-year hemoptysis-free survival rate of up to 79.4% (45). These findings indirectly suggest that NBCA and metallic coils may be effective in preventing hemoptysis recurrence. The omission of these embolic materials in this study may reduce the model’s potential predictive ability. Secondly, the study did not incorporate comprehensive data on subsequent treatments for underlying lung diseases, which could have influenced the hemoptysis recurrence rate. Thirdly, as a retrospective study, selection bias cannot be avoided. Fourthly, the combined model (Model CR) constructed in this study, despite showing excellent predictive performance in the overall population (training set AUC = 0.931, validation set AUC = 0.883) and being able to meet the needs of broad-spectrum clinical risk assessment, fails to fully reveal the unique patterns of hemoptysis recurrence after BAE under a single etiology. It may have limitations in precision prediction for patients with specific etiologies. Fifthly, a total of 170 patients were included in this study, with 35 positive samples. The sample size was sufficient for model construction, but the sample size of some patient subgroups was too small; for example, there were only 6 patients with isolated TB, which may lead to insufficient statistical power. Although the model was internally validated and an H-L test was performed (*p* > 0.05), further large-scale multicenter external validation, especially in cohorts with a higher number of recurrence events, is still needed to fully evaluate its predictive accuracy.

## Conclusion

This study successfully developed and validated a reliable predictive model for forecasting hemoptysis recurrence following BAE. The model demonstrates accurate predictive capability for recurrent hemoptysis in high-risk patients following BAE.

## Data Availability

The original contributions presented in the study are included in the article/Supplementary material, further inquiries can be directed to the corresponding authors.
