# Supplementary material for: Development and validation of a novel nomogram for recurrent hemoptysis after bronchial artery embolization: a population-based cohort study
Source: Front Med (Lausanne). 2025 Dec 19;12:1705253. doi: 10.3389/fmed.2025.1705253 (PMC12757256; doi:10.3389/fmed.2025.1705253)
Supplement: Supplementary file 2 [file Table_2.docx]

**Supplementary Table 2. Inter-observer consistency of radiological indicators**

| **Radiological Indicator** | **Consistency Metric** | **Value (95% CI)** | ***P*** |
| --- | --- | --- | --- |
| Fibrotic scar (categorical) | Cohen’s Kappa | 0.872 (95% CI: 0.821-0.923) | 0.003 |
| Cavity (categorical) | Cohen’s Kappa | 0.891 (95% CI: 0.843-0.939) | 0.001 |
| Pleural thickening (categorical) | Cohen’s Kappa | 0.865 (95% CI: 0.812-0.918) | 0.004 |
| Systemic artery-pulmonary artery fistula (categorical) | Cohen’s Kappa | 0.883 (95% CI: 0.834-0.932) | 0.002 |
| MBAD (continuous, mm) | ICC | 0.792 (95% CI: 0.734-0.841) | < 0.001 |
| NBA (continuous, n) | ICC | 0.834 (95% CI: 0.783-0.874) | < 0.001 |

**Note:** MBAD, maximum bronchial artery diameter; NBA, number of bronchial arteries.
