# Supplementary material for: Development and validation of a novel nomogram for recurrent hemoptysis after bronchial artery embolization: a population-based cohort study
Source: Front Med (Lausanne). 2025 Dec 19;12:1705253. doi: 10.3389/fmed.2025.1705253 (PMC12757256; doi:10.3389/fmed.2025.1705253)
Supplement: Supplementary file 3 [file Table_3.docx]

**Supplementary Table 3. Distribution of independent predictors and recurrence rate across etiological subgroups.**

| **Variable** | **Bronchiectasis** | **TB** | **Malignancy** | **Mixed etiology** | **others** | **Total** | ***P*** |
| --- | --- | --- | --- | --- | --- | --- | --- |
| PLT | 151 (121, 193.5) | 146 (122.75, 185) | 212 (158, 267) | 151 (124, 212.5) | 180 (131, 211) | 161.5 (125, 210.75) | 0.014 |
| CRP | 2.28 (0.81, 9.41) | 8.59 (2.9, 24.03) | 4.55 (1.33, 12.97) | 4.55 (1.14, 19.44) | 3.21 (1.75, 17.25) | 3.32 (1.1, 15.84) | 0.261 |
| Hemoptysis volume |  |  |  |  |  |  | 0.059 |
| Minor | 30 (37.97%) | 3 (50%) | 18 (72%) | 24 (55.81%) | 10 (58.82%) | 85 (50%) |  |
| Moderate | 13 (16.46%) | 2 (33.33%) | 1 (4%) | 6 (13.95%) | 1 (5.88%) | 23 (13.53%) |  |
| Massive | 36 (45.57%) | 1 (16.67%) | 6 (24%) | 13 (30.23%) | 6 (35.29%) | 62 (36.47%) |  |
| MBAD | 3.2 (2.65, 3.6) | 2.3 (2.12, 2.55) | 2.6 (2.4, 3.5) | 3.2 (2.45, 3.6) | 3.2 (2.3, 3.6) | 3.2 (2.4, 3.6) | 0.077 |
| NBA | 2 (2, 3) | 2 (2, 2) | 2 (2, 3) | 2 (2, 3) | 2 (2, 2) | 2 (2, 3) | 0.293 |
| Fibrotic scar | 5 (6.33%) | 1 (16.67%) | 3 (12%) | 10 (23.26%) | 1 (5.88%) | 20 (11.76%) | 0.067 |
| Pleural thickening | 40 (50.63%) | 2 (33.33%) | 17 (68%) | 23 (53.49%) | 11 (64.71%) | 93 (54.71%) | 0.405 |
| Recurrence | 14 (17.72%) | 0 (0%) | 9 (36%) | 8 (18.6%) | 4 (23.53%) | 35 (20.59%) | 0.259 |

**Note:** TB, tuberculosis; mixed etiology refers to the presence of at least two causes among bronchiectasis, TB, and malignant tumors. Others include 1 case of pulmonary abscess, 4 cases of pulmonary infection, 1 case of pneumoconiosis, 3 cases of chronic obstructive pulmonary disease, 4 cases of vascular malformations, and 4 cases with unknown causes. PLT, platelets; CRP, C-reactive protein; MBAD, maximum bronchial artery diameter; NBA, number of bronchial arteries.
