# Supplementary material for: Development and validation of a novel nomogram for recurrent hemoptysis after bronchial artery embolization: a population-based cohort study
Source: Front Med (Lausanne). 2025 Dec 19;12:1705253. doi: 10.3389/fmed.2025.1705253 (PMC12757256; doi:10.3389/fmed.2025.1705253)
Supplement: Supplementary file 4 [file Table_4.docx]

**Supplementary Table 4. Interaction analysis of PLT on hemoptysis recurrence across etiological subgroups**

| Subgroup | Sample size, n (%) | Adjusted OR (95% CI) for PLT | *P* value (subgroup) | *P* value for interaction |  |
| --- | --- | --- | --- | --- | --- |
| All patients | 170 (100.00) | 1.01 (1.00 ~ 1.01) | 0.002 |  |  |
| Etiology |  |  |  |  |  |
| Bronchiectasis | 79 (46.47) | 1.01 (1.00 ~ 1.02) | 0.032 |  |  |
| TB | 6 (3.53) | 1.00 (0.00 ~ Inf) | 1.000 | 0.834 |  |
| Malignancy | 25 (14.71) | 1.00 (0.99 ~ 1.01) | 0.651 |  |  |
| Mixed etiology | 43 (25.29) | 1.01 (1.00 ~ 1.02) | 0.060 |  |  |
| others | 17 (10.00) | 1.01 (0.99 ~ 1.03) | 0.323 |  |  |

**Note:** TB, tuberculosis; mixed etiology refers to the presence of at least two causes among bronchiectasis, TB, and malignant tumors. Others include 1 case of pulmonary abscess, 4 cases of pulmonary infection, 1 case of pneumoconiosis, 3 cases of chronic obstructive pulmonary disease, 4 cases of vascular malformations, and 4 cases with unknown causes. Inf, infinity. The 95% CI of "0.00~Inf" covers all possibilities ranging from "no association (OR = 1)" to "extreme association (OR approaches 0 or infinity)". This result has no practical clinical reference value and cannot be used for inferring the effect of PLT on TB-related hemoptysis recurrence.
